# Decline of coronary heart disease mortality is strongly effected by changing patterns of underlying causes of death: an analysis of mortality data from 27 countries of the WHO European region 2000 and 2013

**DOI:** 10.1007/s10654-020-00699-0

**Published:** 2020-11-28

**Authors:** Susanne Stolpe, Bernd Kowall, Andreas Stang

**Affiliations:** 1 Institute for Medical Informatics, Biometry and Epidemiology, University Hospital Essen, Essen, Germany; 2 Institute for Technical Chemistry, Leibniz-University Hannover, Hannover, Germany; 3 Department of Epidemiology, School of Public Health, Boston University, Boston, USA

**Keywords:** Coronary heart disease mortality, Mortality registry data, Mortality patterns, Causes of death

## Abstract

Mortality rates for coronary heart disease (CHD) experience a longstanding decline, attributed to progress in prevention, diagnostics and therapy. However, CHD mortality rates vary between countries. To estimate whether national patterns of causes of death impact CHD mortality, data from the WHO “European detailed mortality database” for 2000 and 2013 for populations aged ≥ 80 years was analyzed. We extracted mortality rates for total mortality, cardiovascular diseases, neoplasms, dementia and ill-defined causes. We calculated proportions of selected causes of death among all deaths, and proportions of selected cardiovascular causes among cardiovascular deaths. CHD mortality rates were recalculated after re-coding ill-defined causes of death. Association between CHD mortality rates and proportions of CHD deaths was estimated by population-weighted linear regression. National patterns of causes of death were divers. In 2000, CHD was assigned as cause of death in 13–53% of all cardiovascular deaths. Until 2013, this proportion changed between − 65% (Czech Republic) and + 57% (Georgia). Dementia was increasingly assigned as underlying cause of death in Western Europe, but rarely in eastern European countries. Ill-defined causes accounted for between < 1% and 53% of all cardiovascular deaths. CHD mortality rates were closely linked to a countries’ proportion of cardiovascular deaths assigned to CHD (R^2^ = 0.95 for 2000 and 0.99 for 2013). We show that CHD mortality is considerably influenced by national particularities in certifying death. Changes in CHD mortality rates reflect changes in certifying competing underlying causes of death. This must be accounted for when discussing reasons for the CHD mortality decline.

**Electronic supplementary material:**

The online version of this article (doi:10.1007/s10654-020-00699-0) contains supplementary material, which is available to authorized users.

## Introduction

Coronary mortality rates have been declining worldwide—a trend called the “success story of the last 4 decades of the 20th century”; possible causes and implications are in continuing discussion [1]. The mortality rates for coronary heart disease (CHD) though exhibit large differences between countries and over time within a country. Compared to France, reporting the lowest CHD mortality in Europe, CHD mortality rates in Hungary and Slovakia in 2013 were more than eightfold and more than twice as high as in Germany or Sweden [2, 3]. The MONICA project was -among others- motivated by the fact, that in case of declining mortality from cardiovascular diseases “the reasons for these variations are poorly understood” [4].

Country-specific declines in CHD have been attributed to about 90% to effects of primary and secondary prevention, namely risk factor reduction and treatment [4–20]. However, in these analyses the role of divergent and changing patterns in assigning cardiovascular diseases as underlying cause of death has not been taken into account.

To enable valid comparisons of mortality rates by region and calender time, WHO releases instructions on assigning and coding of causes of death. Since 1948, the underlying cause of death has to be recorded as the relevant cause of death, as it is the “most effective public health objective […] to prevent the precipitating cause from operating” [21].

Quality of mortality registries in Europe differs in regard to the proportion of causes of death identified according to the WHO rules. The German mortality registry is of only moderate quality as it comprises about 13% ill-defined deaths, compared to the UK or Finland reporting less than 5% [22]. Ill-defined deaths are causes of death that are not compliant with the WHO definition of underlying cause of death. Ill-defined causes relate to immediate, intermediate, unknown or unspecified causes of death or symptoms present at the time of death. The frequency of ill-defined causes in national mortality registries influences CVD mortality rates and leads to incompatibility [23–25].

In this paper we describe the different and changing patterns of assigning a specific cardiovascular disease as underlying cause of death as well as the frequency of ill-defined cardiovascular causes of death and how this effects CHD mortality rates.

## Materials and methods

We analyzed national mortality data for 2000 and 2013 from the WHO “European detailed mortality database” [26]. This database was available online on the WHO websites (data.euro.who.int/dbmd). In 2018 it was replaced by the European Health Information Gateway (https://gateway.euro.who.int/en/datasets/european-mortality-database/). The database comprises sex and age-specific as well as age-standardized mortality rates by cause of death (three-digit ICD-10) from 1994 to 2014 for 53 countries of the WHO European region. The WHO European region represents all European countries and the countries of the former Soviet Union (such as Kazakhstan, Georgia and the Republic of Moldova) and Israel. Until 2000 and after 2013, however, mortality data for specific causes of death was missing in many countries. Therefore, we selected data from all countries (N = 27) with published CVD mortality rates for 2000 [if not available: 2001 (UK)] and 2013 [if not available: 2012 (DK)] from an offline version that had been downloaded in 2016. Among others, Italy, Portugal and Austria, which could have been expected to be included in this analyses, did not report their mortality data in this time frame.

We selected the age-standardized (ESP 1976) [27] mortality rates per 100,000 person years for populations 35–80+ years and the age-specific mortality rates per 100,000 for age group 80 years and older for the following causes of death: all-cause mortality, cardiovascular diseases (CVD, I00-I99), neoplasms (C00-D48), coronary heart diseases (CHD, I20-I25), cerebrovascular diseases (I60-I69), unspecified causes and symptoms (R00-94, R96-99), essential hypertension (I10), hypertensive heart disease (I11), cardiac arrest (I46), atrial fibrillation and flutter (I48), heart failure (I50), atherosclerosis (I70), vascular dementia (F01), unspecified dementia (F03) and Alzheimers disease (G30). The codes F01, F03 and G30 were summarized as dementia-related diseases.

We calculated the proportion of deaths attributed to cardiovascular diseases, neoplasms, dementia-and unspecified causes and symptoms. Within the subgroup of cardiovascular deaths we calculated the proportion of deaths coded with I20-I25, I60-I69, I10, I46, I48, I50 and I70.

We classified essential hypertension (I10), cardiac arrest (I46), heart failure (I50), atherosclerosis (I70) and deaths coded with R00-R94, R96-R99 as ill-defined causes of death [22]. As these ill-defined causes can mask a CHD as true underlying cause of death we redistributed these deaths to CHD according to a published algorithm. The redistribution algorithm is based on the probability of CHD as the true underlying cause of death in case an ill-defined cause had been assigned originally [28]. The estimated probabilities for CHD being the true cause of death used in this algorithm were 0.90 for deaths from cardiac arrest (I46), 0.77 for deaths from heart failure (I50), 0.70 for essential hypertension (I10), 0.47 for atherosclerosis (I70) and 0.14 for deaths coded with R00-R94, R96-R99. We re-calculated the age-specific and age-standardized CHD-mortality rates after redistribution of ill-defined codes.

We plotted the proportion of CHD deaths among all cardiovascular deaths versus the proportion of ill-defined deaths in cardiovascular deaths for 2000 and 2013 and calculated the explained variance (R^2^) from linear regression weighted by population size.

In the same manner, we visualized the association between the proportion of CHD deaths among all cardiovascular deaths and the reported CHD mortality rate.

We restricted the presented results to the population age group 80 years and older. Results for the age-group ≥ 35 years are similar and available in the supplement.

All analyses were done with SAS 9.3.

### Patient and public involvement

This research did not involve patients or the public as it uses retrospective national mortality data provided by WHO. Neither patients nor the public were involved in the design of this study or the research questions addressed.

## Results

### Cardiovascular disease mortality rates

**Fig. 1:**
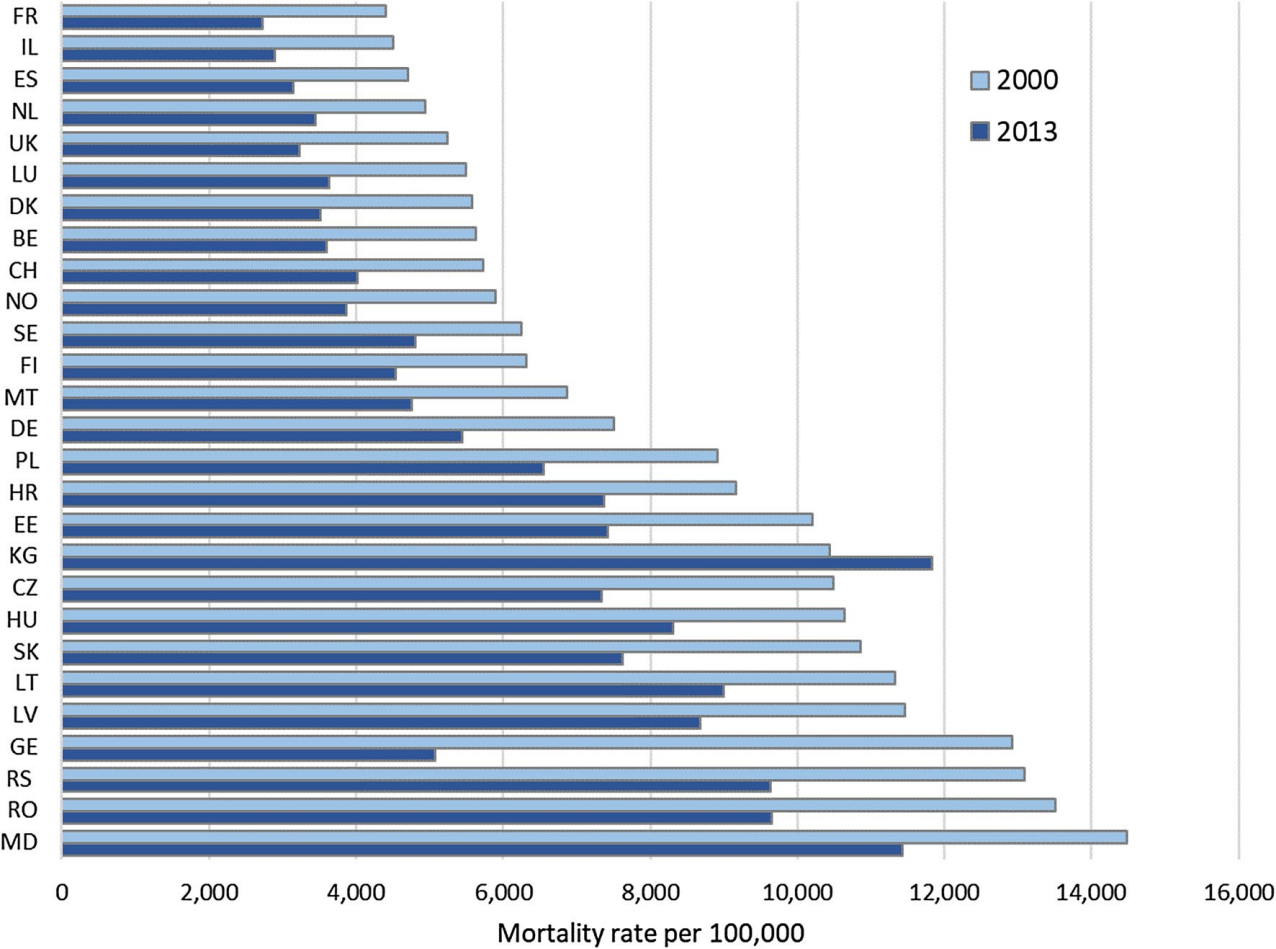
Mortality rate for cardiovascular diseases (CVD, I00-I99) for the population 80 years and older in 2000 and 2013 in the WHO European region. Countries with CVD mortality data available for 2000 (UK: 2001) and 2013 (DK: 2012), ordered by CVD mortality rate 2000. Abbr. *BE* Belgium, *CH* Switzerland, *CZ* Czech Republic, *DE* Germany, *DK* Denmark, *EE* Estonia, *ES* Spain, *FI* Finland, *FR* France, *GE* Georgia, *HR* Croatia, *HU* Hungary, *IL* Israel, *KG* Kyrgyzstan, *LT* Lithuania, *LU* Luxembourg, *LV* Latvia, *MD* Moldova, *MT* Malta, *NL* The Netherlands, *NO* Norway, *PL* Poland, *RO* Romania, *RS* Serbia, *SE* Sweden, *SK* Slovakia, *UK* United Kingdom. Countries of the WHO European Region include: Albania, Armenia, Austria, Azerbaijan, Belarus, Belgium, Bosnia and Herzegovina, Bulgaria, Croatia, Cyprus, Czechia, Denmark, Estonia, Finland, France, Georgia, Germany, Greece, Hungary, Iceland, Ireland, Israel, Italy, Kazakhstan, Kyrgyzstan, Latvia, Lithuania, Luxembourg, Malta, Montenegro, Netherlands, Norway, Poland, Portugal, Republic of Moldova, Romania, Russian Federation, Serbia, Slovakia, Slovenia, Spain, Sweden, Switzerland, Tajikistan, The former Yugoslav Republic of Macedonia, Turkey, Turkmenistan, Ukraine, United Kingdom, Uzbekistan

Cardiovascular disease mortality rates for ages 80 years and more declined in all selected countries-but Kyrgyzstan-between 2000 and 2013. The size of cardiovascular mortality rates and the absolute decline differed strongly. In 2013, mortality rates for CVD ranged from 2700/100,000 in France to 12,000/100,000 in Kyrgyzstan (Fig. 1). Absolute decline was −1500/100,000 in Sweden and the Netherlands, but −4000/100,000 in Romania and even −8000/100,000 in Georgia. Relatively, CVD mortality rates in Moldavia and Lithuania declined least (21%), France and the UK experienced a decline of 38%, Georgia of 61%.

### Coronary heart disease mortality rates

**Fig. 2:**
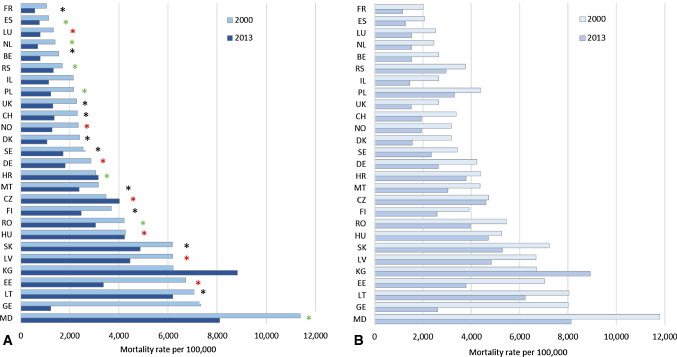
Mortality rates for coronary heart disease (CHD, ICD10: I20-I25) in the population 80 years and older in 2000 and 2013 in the WHO European Region. Countries with CHD mortality data available for 2000 (UK: 2001) and 2013 (DK: 2012). Countries ordered by CHD mortality rate 2000. Panel A: as reported. Panel B: after redistribution and recoding of ill-defined deaths (ICD-10: I10, I46, I50, I70 and R-codes). Coding method: black asterisk automated coding, green asterisk manual coding, red asterisk change between 2000 and 2013 from manual to automated coding (https://ec.europa.eu/eurostat/cache/metadata/en/hlth_cdeath_esms.htm. Access 14.9.2020). BE and DE implemented automated coding only in some parts of the country. Information on mortality statistics in the Republic of Moldova indicate manual coding. No information available for Israel, Georgia and Kazakhstan. Abbr.: *BE* Belgium, *CH* Switzerland, *CZ* Czech Republic, *DE* Germany, *DK* Denmark, *EE* Estonia, *ES* Spain, *FI* Finland, *FR* France, *GE* Georgia, *HR* Croatia, *HU* Hungary, *IL* Israel, *KG* Kyrgyzstan, *LT* Lithuania, *LU* Luxembourg, *LV* Latvia, *MD* Moldova, *MT* Malta, *NL* The Netherlands, *NO* Norway, *PL* Poland, *RO* Romania, *RS* Serbia, *SE* Sweden, *SK* Slovakia, *UK* United Kingdom

Relative decline in mortality rates for CHD from 2000 to 2013 ranged from 33% (Spain, Finland) to 46% (Norway, France) in Western Europe and from 15% in Lithuania to 83% in Georgia in eastern Europe. Largest absolute decline was seen in Georgia. In Hungary, Croatia, the Czech Republic and Kyrgyzstan CHD mortality rates remained either unchanged or increased (Fig. 2a).

### CHD mortality after redistribution of ill-defined causes of death

After redistribution of deaths attributed to the ill-defined causes essential hypertension (ICD-10 I10), cardiac arrest (I46), heart failure (I50), atherosclerosis (I70) and R-coded deaths (R00-R99), the CHD mortality rates increased in relation to the proportion and type of ill-defined causes of death. While the increase was low (0.02–4.5%) in Moldova, Lithuania, Latvia and Finland, the recalculated rates exceeded the reported rates by 35–90% in most countries in 2000 and 2013. In 2000, redistributed mortality rates were more than twice as high for Poland and Serbia, in 2013 this applied additionally for France and the Netherlands (Fig. 2b). CHD mortality rates more than doubled after redistribution compared to the reported mortality rates.

### Proportion of deaths attributed to cardiovascular diseases

**Fig. 3:**
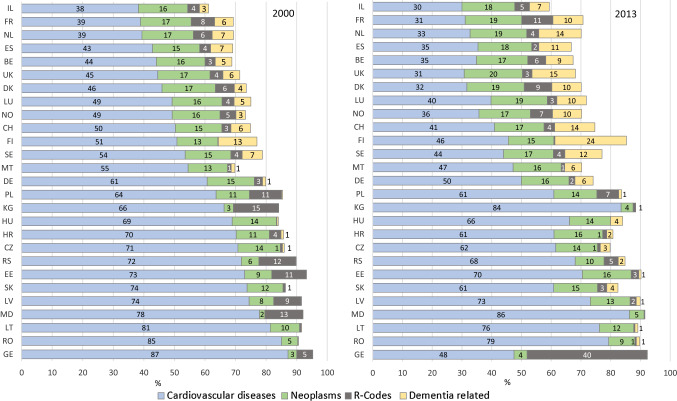
Among all deaths, proportions of deaths from cardiovascular diseases (CVD, I00-I99), neoplasms (C00-D48), R-coded and dementia related (F01, F03, G30) deaths in the population 80 years and older in the WHO European Region in 2000 (left panel) and 2013 (right panel). Countries ordered by proportion of deaths from cardiovascular diseases in 2000. Numbers within bars represent the proportions; proportions less than 0.5 are not given. Abbr.: *BE* Belgium, *CH* Switzerland, *CZ* Czech Republic, *DE* Germany, *DK* Denmark, *EE* Estonia, *ES* Spain, *FI* Finland, *FR* France, *GE* Georgia, *HR* Croatia, *HU* Hungary, *IL* Israel, *KG* Kyrgyzstan, *LT* Lithuania, *LU* Luxembourg, *LV* Latvia, *MD* Moldova, *MT* Malta, *NL* The Netherlands, *NO* Norway, *PL* Poland, *RO* Romania, *RS* Serbia, *SE* Sweden, *SK* Slovakia, *UK* United Kingdom

The proportion of deaths attributed to CVD among all deaths differed between countries and decreased over time. In 2000, CVD accounted for about 38% of all deaths in Israel, France and the Netherlands (in 2013: 30–33%), but for 85% in Romania (2013: 79%) and 87% in Georgia (2013: 48%) (Fig. 3).

### Proportion of deaths attributed to dementia-related diseases (ICD-10: F01, F03, G30)

The proportion of deaths attributed to dementia-related diseases increased from 2000 to 2013 and revealed an east–west divide. While this proportion in 2000 was far below 1% in Eastern Europe, it amounted up to 13% in Finland. In 2013, in western European countries 10–24% of all deaths were attributed to dementia. In East-Europe, this proportion remained below 4% (Fig. 3).

### Proportion of deaths attributed to symptoms and unspecified causes of death (ICD-10 R-codes)

ICD-10 codes for “symptoms, signs and abnormal clinical and laboratory findings”—including ill-specified and unknown causes of mortality (R00-94, R96-99)—had been assigned in 0.2% of all deaths in Finland, but in about 11% in Poland, Serbia and Estonia in 2000. In 2013, 10% of all deaths were R-coded in France, and 40% in Georgia compared to less than 1% of all deaths in Finland, Hungary, Lithuania and the Czech Republic (Fig. 3).

### Cardiovascular deaths attributed to CHD

**Fig. 4:**
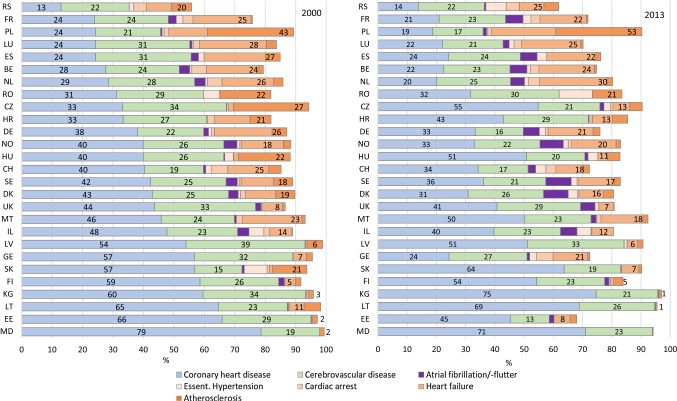
Among all cardiovascular deaths, proportion of deaths from selected cardiovascular diseases in populations 80 years and older in the WHO European Region in 2000 (left panel) and 2013 (right panel). Ill-defined cardiovascular causes (essential hypertension, cardiac arrest, heart failure and atherosclerosis) are indicated by reddish filling. Numbers represent the respective proportions. Numbers in the reddish bar indicate the proportion of all selected ill-defined causes combined; proportions less 0.5 are not given. Countries are ordered according to their proportion of deaths from coronary heart disease in 2000. Abbr.: *BE* Belgium, *CH* Switzerland, *CZ* Czech Republic, *DE* Germany, *DK* Denmark, *EE* Estonia, *ES* Spain, *FI* Finland, *FR* France, *GE* Georgia, *HR* Croatia, *HU* Hungary, *IL* Israel, *KG* Kyrgyzstan, *LT* Lithuania, *LU* Luxembourg, *LV* Latvia, *MD* Moldova, *MT* Malta, *NL* The Netherlands, *NO* Norway, *PL* Poland, *RO* Romania, *RS* Serbia, *SE* Sweden, *SK* Slovakia, *UK* United Kingdom

In 2000, 13% of the cardiovascular deaths were attributed to CHD in Serbia—compared to 66% in Estonia. The proportion of CHD as cause of a cardiovascular death increased in Slovakia (+ 7%-points), Hungary (+ 11%-points) and the Czech Republic (+ 22%-points). It decreased in all other countries, especially in Georgia (− 33%-points), Estonia (− 21%-points) and the Netherlands (− 9%-points) (Fig. 4).

### Cardiovascular deaths attributed to ill-defined cardiovascular causes

In 2000, the proportion of cardiovascular deaths attributed to ill-defined causes of death (ICD-10: I10, I46, I50, I70) ranged from 2% in Estonia to 42% in Poland (Fig. 4). Until 2013, this proportion increased in Poland, Serbia, Georgia, Spain and the Netherlands, but declined markedly in other countries. Heart failure (I50) was the most often reported ill-defined cause of death in Europe. In 2000, the proportion of heart failure among all CVD deaths ranged from below 2% in the Czech Republic, Slovakia, Latvia and Estonia to about 20% in the Netherlands and Spain. In 2013, in Poland heart failure was assigned as underlying cause of death in 22% of all cardiovascular deaths. Atherosclerosis was by far the most common ill-defined cardiovascular cause of death in 2000 in Poland and the Czech Republic with an increasing proportion until 2013 in Poland. In Serbia, Georgia, Slovakia and Switzerland deaths from cardiac arrest or essential hypertension accounted for 8–11% of all CVD deaths in 2013.

### Cardiovascular deaths attributed to atrial fibrillation and flutter

The proportion of atrial fibrillation and flutter (I48) as underlying cause of death among all CVD deaths increased in the western European countries until 2013. While its proportion among all deaths from CVD did not exceed 2% in most European countries in 2000, it more than doubled in many western-European countries until 2013 (Fig. 4).

### Association between the CHD mortality rate and the proportion of deaths attributed to CHD

**Fig. 5:**
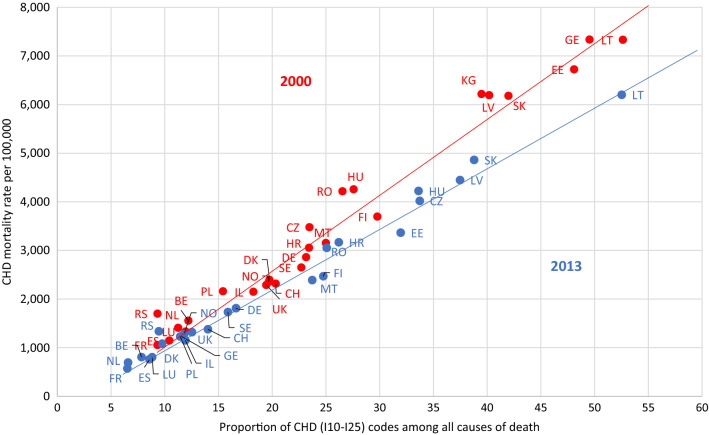
Association of the proportion of coronary heart disease among all causes of death and the coronary heart disease mortality rate in populations 80 years and older in the WHO European region in 2000 (red dots) and 2013 (blue dots). Explained variances of population-weighted linear regression: R^2^ = 0.95 for 2000 and R^2^ = 0.99 for 2013. (Not shown due to better readability of the graph: data for MD in 2000: 62; 11.385 and 2013: 61; 8.111 and for KG in 2013: 62; 8.826). Abbr.: *BE* Belgium, *CH* Switzerland, *CZ* Czech Republic, *DE* Germany, *DK* Denmark, *EE* Estonia, *ES* Spain, *FI* Finland, *FR* France, *GE* Georgia, *HR* Croatia, *HU* Hungary, *IL* Israel, *KG* Kyrgyzstan, *LT* Lithuania, *LU* Luxembourg, *LV* Latvia, *MD* Moldova, *MT* Malta, *NL* The Netherlands, *NO* Norway, *PL* Poland, *RO* Romania, *RS* Serbia, *SE* Sweden, *SK* Slovakia, *UK* United Kingdom

The mortality rate for CHD is strongly associated with the proportion of deaths attributed to CHD in 2000 and 2013 (Fig. 5). In most countries, a decrease in CHD mortality rate from 2000 to 2013 went in hand with a similarly declining proportion of deaths attributed to CHD. However, different patterns were visible. In Lithuania, Latvia and Slovakia, CHD mortality rate decreased while the proportion of CHD deaths remained at a similar level; in Serbia, Hungary and Croatia, CHD mortality remained stable while CHD proportion among all deaths decreased.

### Association between the proportion of deaths from CHD and the proportion of ill-defined causes

**Fig. 6:**
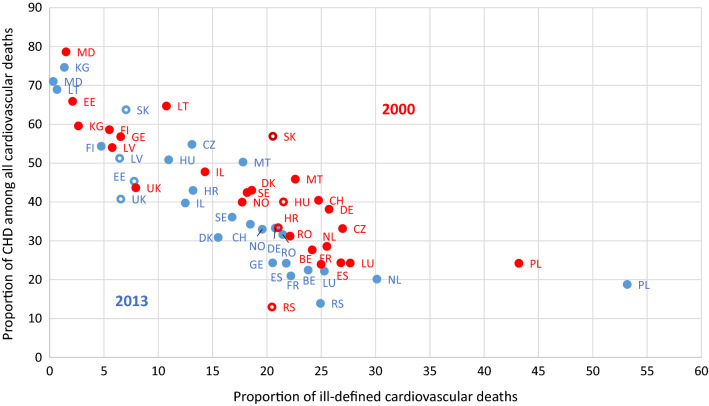
Association of the proportion of coronary heart disease among all causes of death and the coronary heart disease mortality rate in populations 80 years and older in the WHO European region in 2000 and 2013. Explained variances: R^2^ = 0.95 for 2000 and R^2^ = 0.99 for 2013

Among cardiovascular deaths, the higher the proportion of ill-defined deaths, the lower is on average the proportion of cardiovascular deaths attributed to CHD. However, in 2000, Serbia (13%), Croatia (34%), Hungary (40%) and Slovakia (57%) had different proportions of deaths from CHD while the reporting similar proportions of ill-defined cardiovascular causes (about 22%). In 2013, Slovakia, Latvia, Estonia and the UK differed considerably in the proportion of cardiovascular deaths attributed to CHD, but all had a similar proportion of ill-defined cardiovascular deaths (Fig. 6).

## Discussion

In the WHO European region, CHD mortality rates for the age group 80+ as well as for younger ages (see supplementary files) are differentially influenced by changing national patterns of certifying the underlying cause of death. Patterns in assigning underlying causes of death comprise varying proportions of ill-defined causes of death. If these national preferences are not accounted for, conclusions about coronary heart disease morbidity, and about the reasons for the ongoing decline of coronary heart disease mortality can only be drawn very cautiously using mortality registry data.

The proportion of cardiovascular deaths attributed to CHD varied broadly in the WHO European region and predicted the reported CHD mortality rate to a great extent (Fig. 5). The proportion of cardiovascular deaths from CHD was, in turn, affected by the proportion of ill-defined cardiovascular deaths. In 2013, the proportion of deaths from CHD among all deaths was 6.5% in France and the Netherlands, 13% in the UK, 24% in Finland and 61% in Moldova. These differences most likely reflect divergent underlying distributions of competing cardiovascular and non-cardiovascular diseases certified as underlying causes of death. In most European countries, the distribution of certified causes of death changed considerably over time. The proportion of cardiovascular causes of death steadily declined. Consequently, mortality rates for CHD declined. Although this seems clear and has to be expected, it has not been considered so far in any analyses on the reasons for the decline in CHD mortality. During the last centuries, risk factor control and therapeutic progress were claimed equally responsible for the reduction in CHD mortality rates in many country-specific analyses of population-based epidemiologic cohorts [12, 13, 18] and national mortality registries [5–11, 15–17, 19, 20, 29–32].

### Influence of coding method and ICD10 edition in use on cause-specific mortality rates

Among all information on a death certificate, the underlying cause of death is identified and coded according to the ICD10 coding rules [21] in the national statistical office. ICD-10 coding rules are updated regularly. In the 2010 edition, coders were advised to prefer dementia over cerebrovascular disease as cause of death in case dementia was reported as caused by cerebrovascular diseases [33]. This rule had not been part of ICD10 volume 2 in 2003 [34]. However, not all countries implement all ICD10 editions, some countries implement them with delay. Therefore, national differences in the coding due to the ICD10 editions in use could impact national patterns of causes of death. Moreover, implementing the ICD10 coding rules in automatic coding systems should lead to a more uniform application compared to manual coding. An analysis of Dutch death certificates showed that after switching from manual to automated coding in the Netherlands, dementia was more often coded as underlying cause of death using automated coding [35]. In our data though, the proportion of deaths from CHD and dementia changed independently of the coding method used in a country (Fig. 2). The national patterns of causes of death and the CHD mortality rates in 2000 and 2013 did not show any systematic differences that could be explained by the (changed) coding system.

### Experience in certifying death is lacking

Registry information using underlying cause of death information relies more or less solely on the experience and attention of the certifying physicians. Measures for quality control of death certification information are rare and autopsy rates low [36]. Physicians certifying death are usually neither trained nor experienced. The WHO definition of ‘cause of death’ is rarely familiar to them [37]. This lack of knowledge and experience directly influences cause-specific mortality rates. In France, contrasting to Finland [38], a considerable underestimation of CHD mortality was found in the national registry [39]. However, continuing education and training can improve the quality of death certifications considerably [40–42] in order to “saving lives through certifying death” [43].

Following the WHO-definition—which may often even contradict medical thinking or “may appear wrong or questionable from a purely medical perspective” [21] —the expected mortality rate for heart failure should be close to zero. According to ICD10 Vol 2 edition 2010, heart failure (I50) could be coded as underlying cause of death when causing pulmonary edema (J81) or atherosclerosis (I70) only [33]. While mortality rates for heart failure were close to zero in Romania and Moldova, they surpassed the mortality rates for CHD in the Netherlands and Poland in 2013. Mortality rates for heart failure and their changes and trends have been reported from many countries and discussed in scientific journals and publications from cardiological associations [44, 45].

### National preferences in determining causes of death lead to diverse distributions of causes of death

Certifying physicians are influenced by national and personal preferences in identifying the underlying cause of death. In Georgia, the proportion of R-coded deaths increased strongly between 2000 and 2013. This change was accompanied by a steep decline of the proportion of deaths attributable to CVD and CHD and a decrease in the CHD mortality rate by 83%. In Serbia, 31% of all cardiovascular deaths were attributed to cardiomyopathy (ICD-10 I42), a cause of death rarely certified elsewhere. In Estonia, Switzerland, and France, hypertensive heart disease (I11) was certified more frequently than average. In Finland, every 4th death in the 80+ year old population was attributed to dementia, a cause of death that has been rarely certified as underlying cause of death in other countries. These country-specific particularities limited differentially the proportion of cardiovascular deaths left over as potential CHD deaths.

### Preferential coding of dementia according to WHO affects mortality rate for CHD

WHO coding rules promote the coding of dementia as underlying cause of death in presence of selected other causes of death listed on a death certificate [21]. This ruling might have been motivated to highlight the increasing public health relevance of dementia in high-and middle-income countries [43]. However, as multi-morbidity is highly prevalent in older age, it is unclear whether the preferential coding of dementia as underlying cause of death is still proportionate. Physicians’ awareness for dementia is equally rising, increasing the probability of certifying dementia as cause of death [46]. In 2000, dementia ranked 93th in the national mortality registry among the most frequent causes of death in Germany for all ages, in 2017 it was the fourth most frequently registered cause of death. Mortality rates for dementia and CHD have direct impact on each other, as they are competing causes of death in older ages. Rising mortality rates for dementia lead to declining mortality rates for CHD.

### Regional differences in the availability of innovative treatment regimens for cardiovascular diseases impacts CHD mortality rates

Progress in treatment procedures results in newly emerging competing causes of death, such as atrial fibrillation and flutter (ICD-10 I48). As societies grow older, more people potentially are at risk of being diagnosed atrial fibrillation and flutter. Atrial fibrillation and flutter ranked 4th among all main diagnoses for hospital admissions in 2016 in Germany [47]. It ranked 11th in absolute number of deaths, compared to rank 31 in 2000. The increasing proportion of deaths from atrial fibrillation and flutter in the aged population of western European countries (Fig. 4) may reflect availability and utilization of modern treatment procedures such as catheter ablations [48] as well as the physicians awareness for this condition [44]. However, research on causes of death in patients with atrial fibrillation and flutter identified other cardiovascular and non-cardiovascular diseases as underlying cause of death [49, 50].

### Inexplicable findings regarding CHD mortality rates explained

According to US mortality registry data, CHD mortality seems to decline at slower pace in younger age groups (35–54 years). A finding the authors could not explain [11]. In the light of our analysis, we suggest that the CHD mortality rate in younger age groups is less affected and distorted by concomitant diseases that could be assigned as underlying cause of death instead of CHD. Frequent concomitant diseases and competing causes of death as dementia, atrial fibrillation and flutter or heart failure evolve predominantly in higher age, impacting negatively CHD mortality. Therefore, the effect of preventive or curative measures on the mortality rates for CHD should be estimated more validly in the younger age groups.

In 1993, the French paradox emerged [29], when an analysis of CHD mortality rates in France and Finland described a similar risk profile regarding dietary factors in the populations, but found a fourfold CHD mortality rate in Finland compared to France. These findings should be re-evaluated in light of the highly divergent patterns of diseases assigned as underlying causes of death in Finland and France that might overrule the influence of dietary habits on CHD mortality.

### Strengths and limitations of the study

Using the “European detailed mortality database” we were able to analyze information on specific cardiovascular and non-cardiovascular causes of death coded by ICD10 at the three digit-level. These data from official national mortality registries spanning a period of 14 years, represent a unique source for exploring the divers and changing patterns of underlying causes of death for most of the European countries.

As many ill-defined ICD-10 codes have to be identified on a four-digit level we could not account for all ill-defined CVD deaths in our analysis. In case of atherosclerosis, we took ICD-10 code I70 as proxy for the ill-defined cause I70.9 (other and unspecified atherosclerosis) as in Germany > 90% of deaths from atherosclerosis (I70) had been actually coded with I70.9. We do not claim that our redistribution of ill-defined deaths results in true mortality rates for CHD. However, the impact of differing prevalence of ill-defined causes of death on CHD mortality rates gets clearly visible. The redistributed rates still underestimate the true CHD mortality.

Using the most detailed, publicly available, information on causes of death in Europe, we are the first to our knowledge to describe in depth the national particularities in certifying death, which particularly result in distorted declining CHD mortality rates. This distortion prevents valid comparisons of CHD mortality rates between countries and points of time.

## Conclusion

National particularities in certifying death revealed diverse patterns of diseases identified as underlying cause of death. These patterns changed over time and especially impacted CHD mortality rates. Due to the differential use of ill-defined and competing causes of death in certifying, national registry data for coronary heart disease mortality are distorted and lead to underestimation of the true underlying morbidity to a varying, unknown extent. Comparisons of CHD mortality rates as reported in national mortality registries between countries and over time are highly compromised. Therefore, the discussion on the factors causing the longstanding decline in CHD mortality, such as progress in preventive and curative interventions, cannot be validly based on mortality registry data. Data from population-based epidemiologic cohorts with validated cause of death information should be used instead. In general, redistribution and recalculation of ill-defined causes of death should be considered before reporting and comparing CHD mortality.

## Electronic supplementary material

Below is the link to the electronic supplementary material.

Supplementary Material (PDF 535 kb)

## Data Availability

The European Detailed Mortality database has been relaunched by WHO in 2018. The in-depth mortality information we exploited for our study is no longer available (https://gateway.euro.who.int/en/). The European Detailed Mortality database (last update 2016) is though available in as downloaded in 2016 from the author at Susanne.stolpe@uk-essen.de. None.
