# Supplementary material for: Decline of coronary heart disease mortality is strongly effected by changing patterns of underlying causes of death: an analysis of mortality data from 27 countries of the WHO European region 2000 and 2013
Source: Eur J Epidemiol. 2020 Nov 28;36(1):57–68. doi: 10.1007/s10654-020-00699-0 (PMC7847455; doi:10.1007/s10654-020-00699-0)
Supplement: Supplementary file 1 — Supplementary Material (PDF 535 kb) [file 10654_2020_699_MOESM1_ESM.pdf]

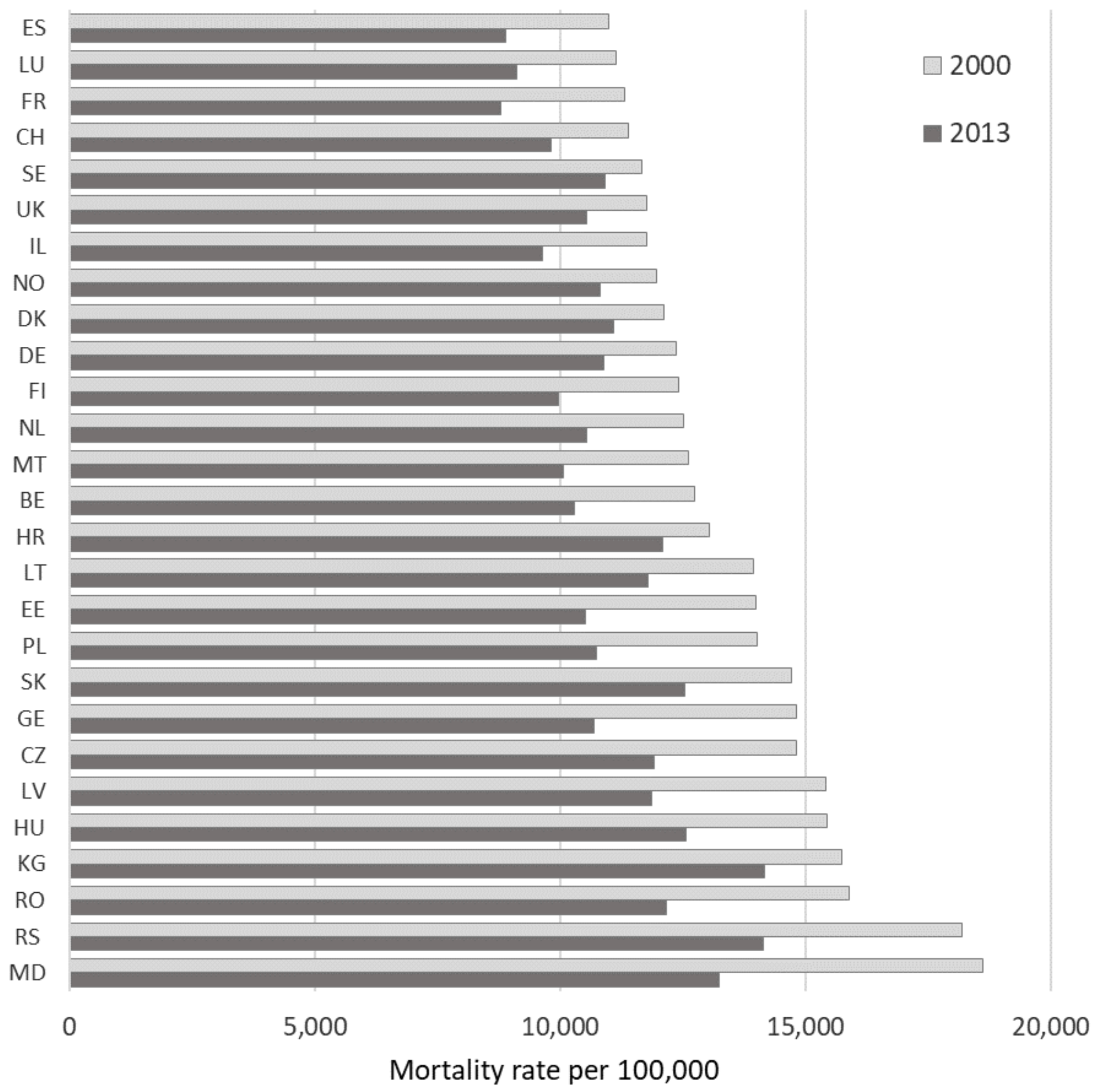

Table S1a) Total mortality rates per 100,000 in the population 80 years and older in 2000 (dark bars) and 2013 (light bars) in the WHO European Region. Countries ordered by total mortality rate 2000.

Abbr.: BE: Belgium, CH: Switzerland, CZ: Czech Republic, DE: Germany, DK: Denmark, EE: Estonia, ES: Spain, FI: Finland, FR: France, GE: Georgia, HR: Croatia, HU: Hungary, IL: Israel, KG: Kyrgyzstan, LT: Lithuania, LU: Luxembourg, LV: Latvia, MD: Moldova, MT: Malta, NL: The Netherlands, NO: Norway, PL: Poland, RO: Romania, RS: Serbia, SE= Sweden, SK: Slovakia, UK: United Kingdom.

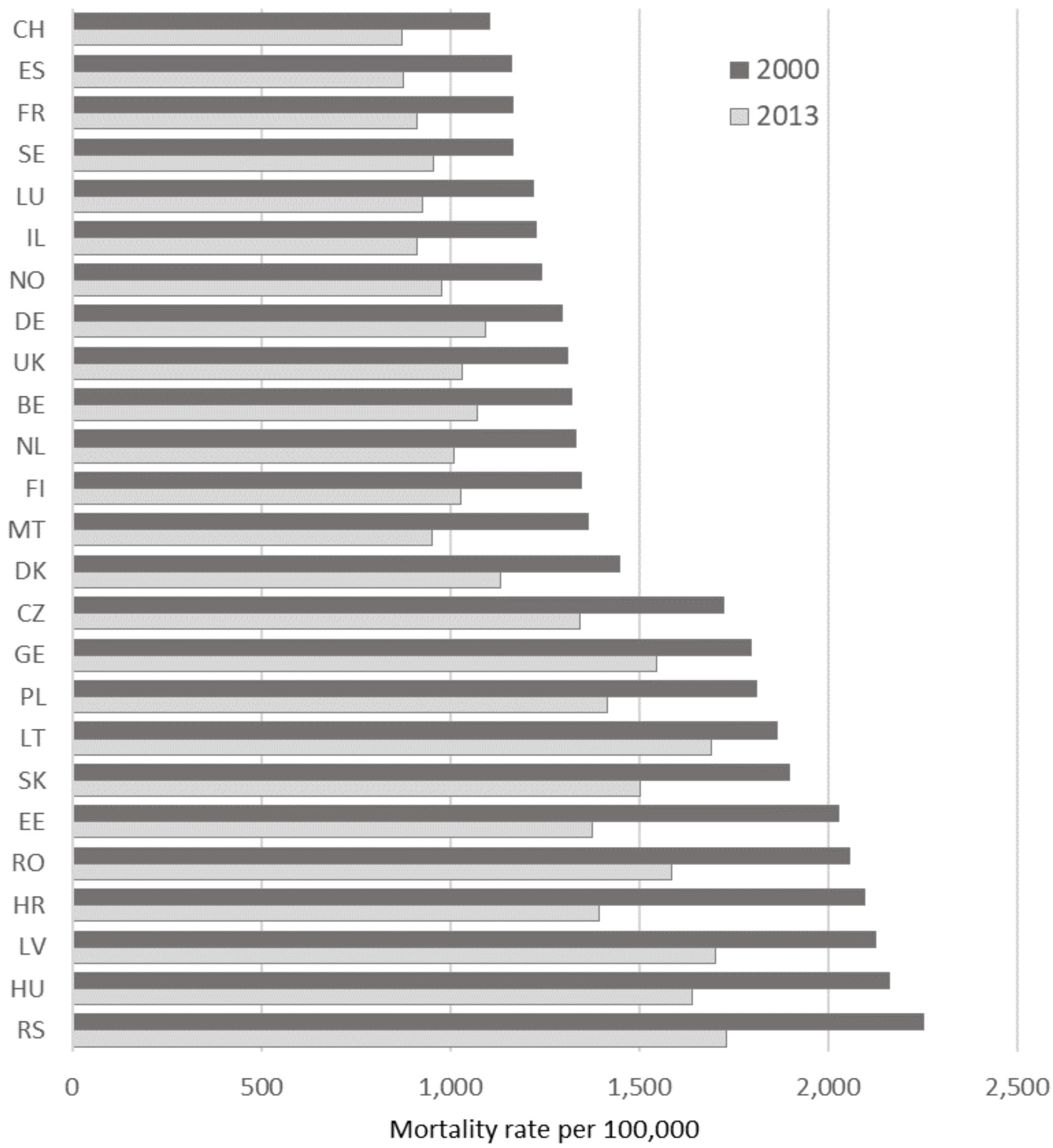

Table S1b) Total age-standardized (ESP76) mortality rates per 100,000 in the population ≥35years in 2000 (dark bars) and 2013 (light bars) in the WHO European Region. Countries ordered by total mortality rate 2000.

Abbr.: BE: Belgium, CH: Switzerland, CZ: Czech Republic, DE: Germany, DK: Denmark, EE: Estonia, ES: Spain, FI: Finland, FR: France, GE: Georgia, HR: Croatia, HU: Hungary, IL: Israel, KG: Kyrgyzstan, LT: Lithuania, LU: Luxembourg, LV: Latvia, MD: Moldova, MT: Malta, NL: The Netherlands, NO: Norway, PL: Poland, RO: Romania, RS: Serbia, SE= Sweden, SK: Slovakia, UK: United Kingdom.

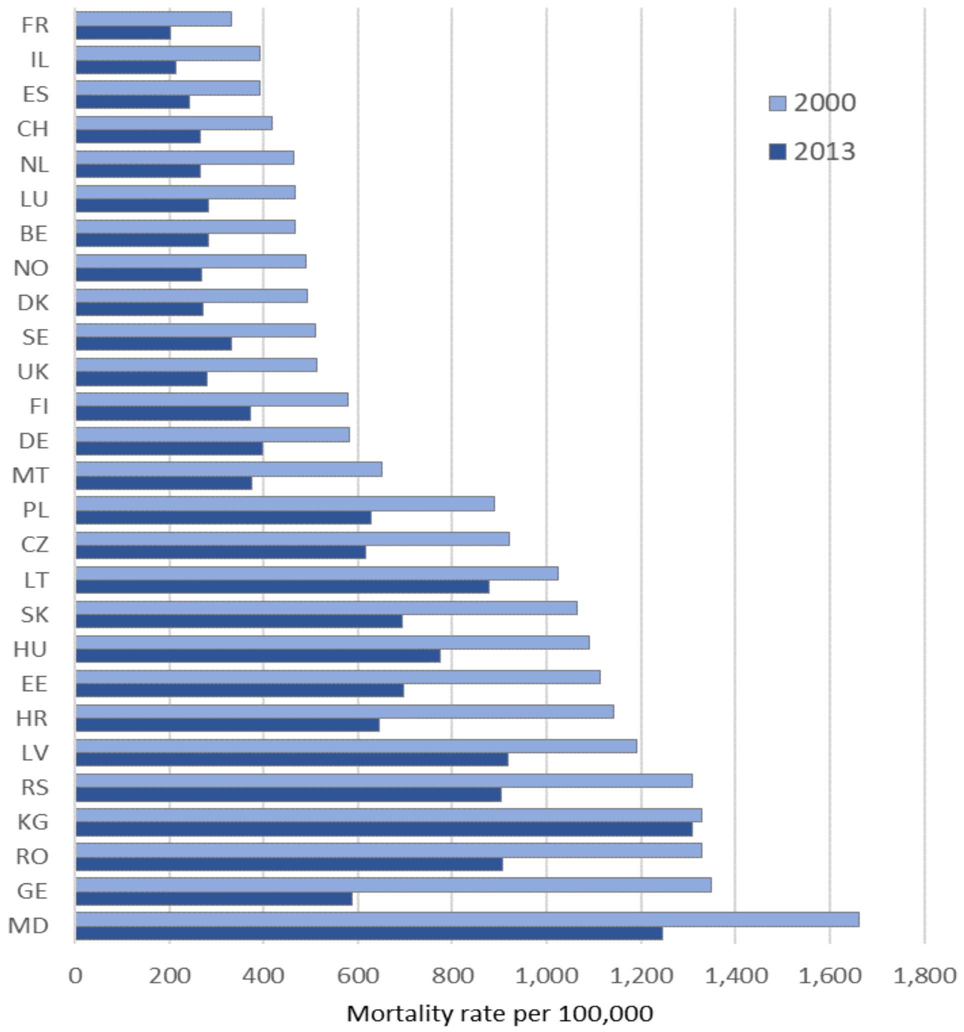

Table S2:

Age standardized (ESP76) mortality rates for cardiovascular diseases (CVD, ICD10: I00-I99) in the population ≥35years in 2000 and 2013 in the WHO European Region (countries ordered by CHD mortality rate 2000).

Abbr.: BE: Belgium, CH: Switzerland, CZ: Czech Republic, DE: Germany, DK: Denmark, EE: Estonia, ES: Spain, FI: Finland, FR: France, GE: Georgia, HR: Croatia, HU: Hungary, IL: Israel, KG: Kyrgyzstan, LT: Lithuania, LU: Luxembourg, LV: Latvia, MD: Moldova, MT: Malta, NL: The Netherlands, NO: Norway, PL: Poland, RO: Romania, RS: Serbia, SE= Sweden, SK: Slovakia, UK: United Kingdom.

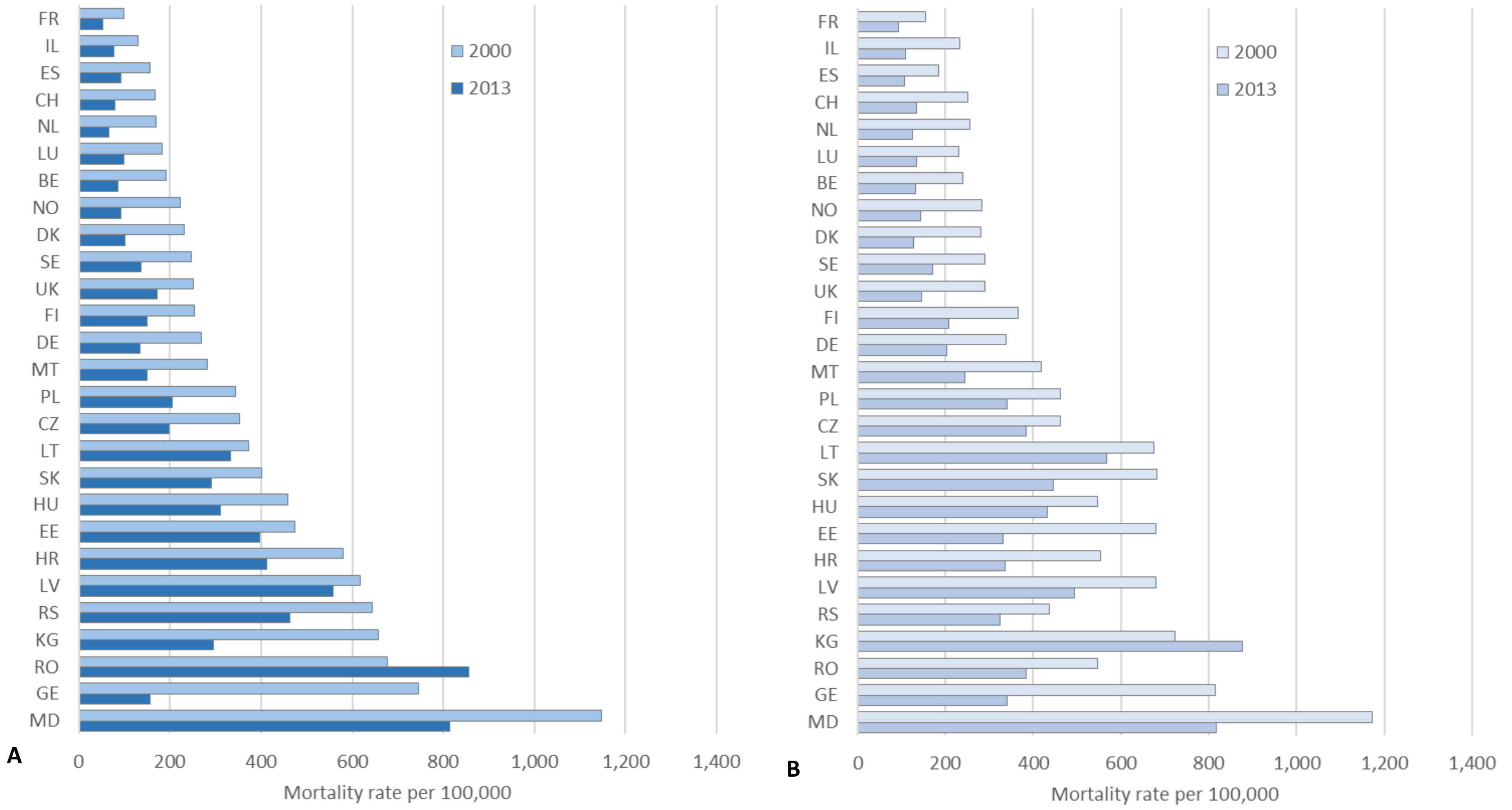

Table S3: Age standardized (ESP76) mortality rates for coronary heart disease (CHD, ICD10: I20-I25) in the population ≥35years in 2000 and 2013 in the WHO European Region (countries ordered by CHD mortality rate 2000). Panel A: as reported; Panel B: after redistribution and recoding of ill-defined deaths (ICD-10: I10, I46, I50, I70 and R-codes).

Abbr.: BE: Belgium, CH: Switzerland, CZ: Czech Republic, DE: Germany, DK: Denmark, EE: Estonia, ES: Spain, FI: Finland, FR: France, GE: Georgia, HR: Croatia, HU: Hungary, IL: Israel, KG: Kyrgyzstan, LT: Lithuania, LU: Luxembourg, LV: Latvia, MD: Moldova, MT: Malta, NL: The Netherlands, NO: Norway, PL: Poland, RO: Romania, RS: Serbia, SE= Sweden, SK: Slovakia, UK: United Kingdom.

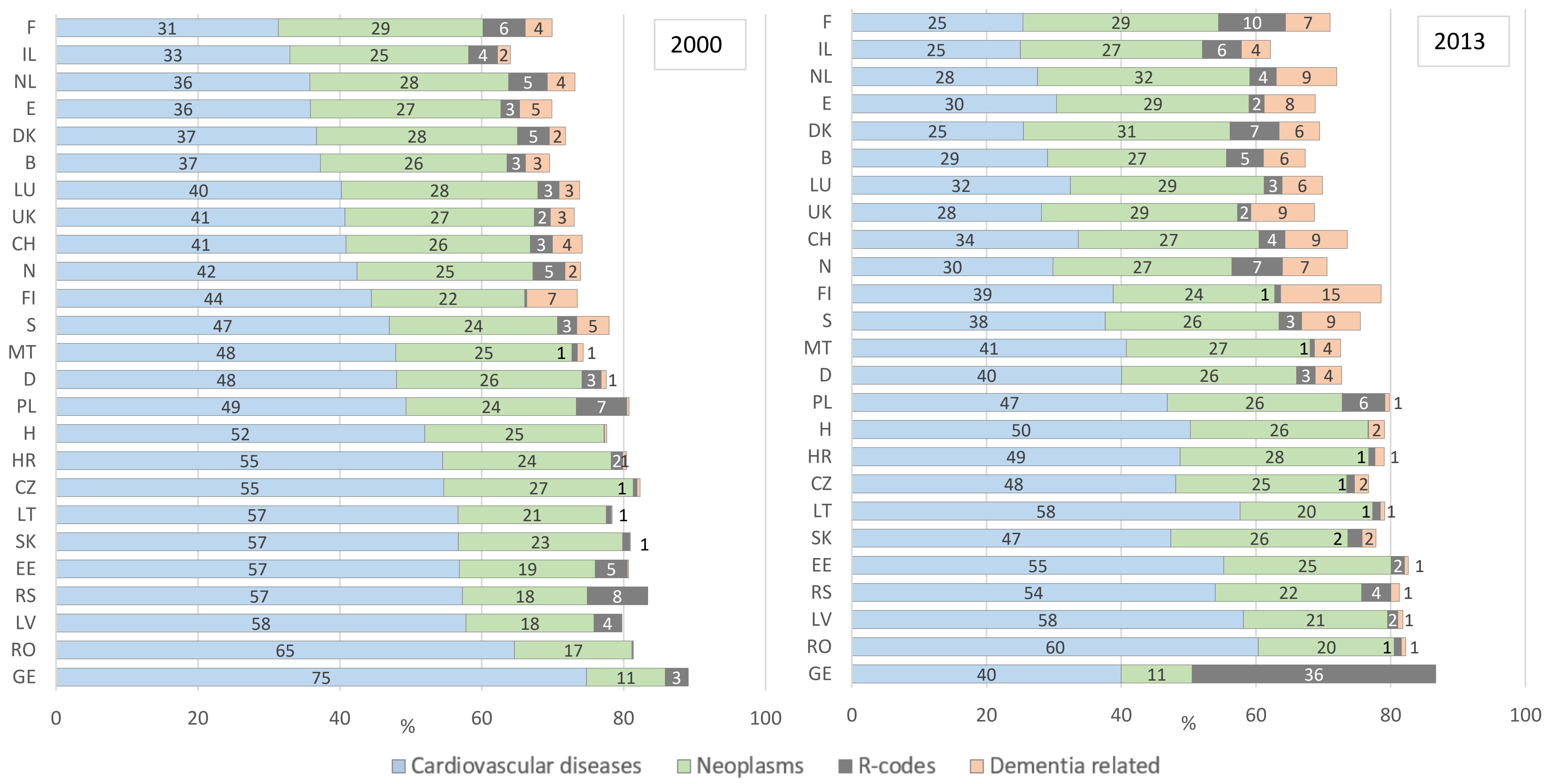

Fig S4: Among all deaths, proportions of deaths from cardiovascular diseases (CVD, I00-I99), neoplasms (C00-D48), R-coded and dementia related (F01, F03, G30) deaths in the population ≥35 years in the WHO European Region in 2000 (left panel) and 2013 (right panel). Countries ordered by proportion of deaths from cardiovascular diseases in 2000. Numbers within bars represent the proportions; proportions less than 0.5 are not given.

Abbr.: BE: Belgium, CH: Switzerland, CZ: Czech Republic, DE: Germany, DK: Denmark, EE: Estonia, ES: Spain, FI: Finland, FR: France, GE: Georgia, HR: Croatia, HU: Hungary, IL: Israel, KG: Kyrgyzstan, LT: Lithuania, LU: Luxembourg, LV: Latvia, MD: Moldova, MT: Malta, NL: The Netherlands, NO: Norway, PL: Poland, RO: Romania, RS: Serbia, SE= Sweden, SK: Slovakia, UK: United Kingdom.

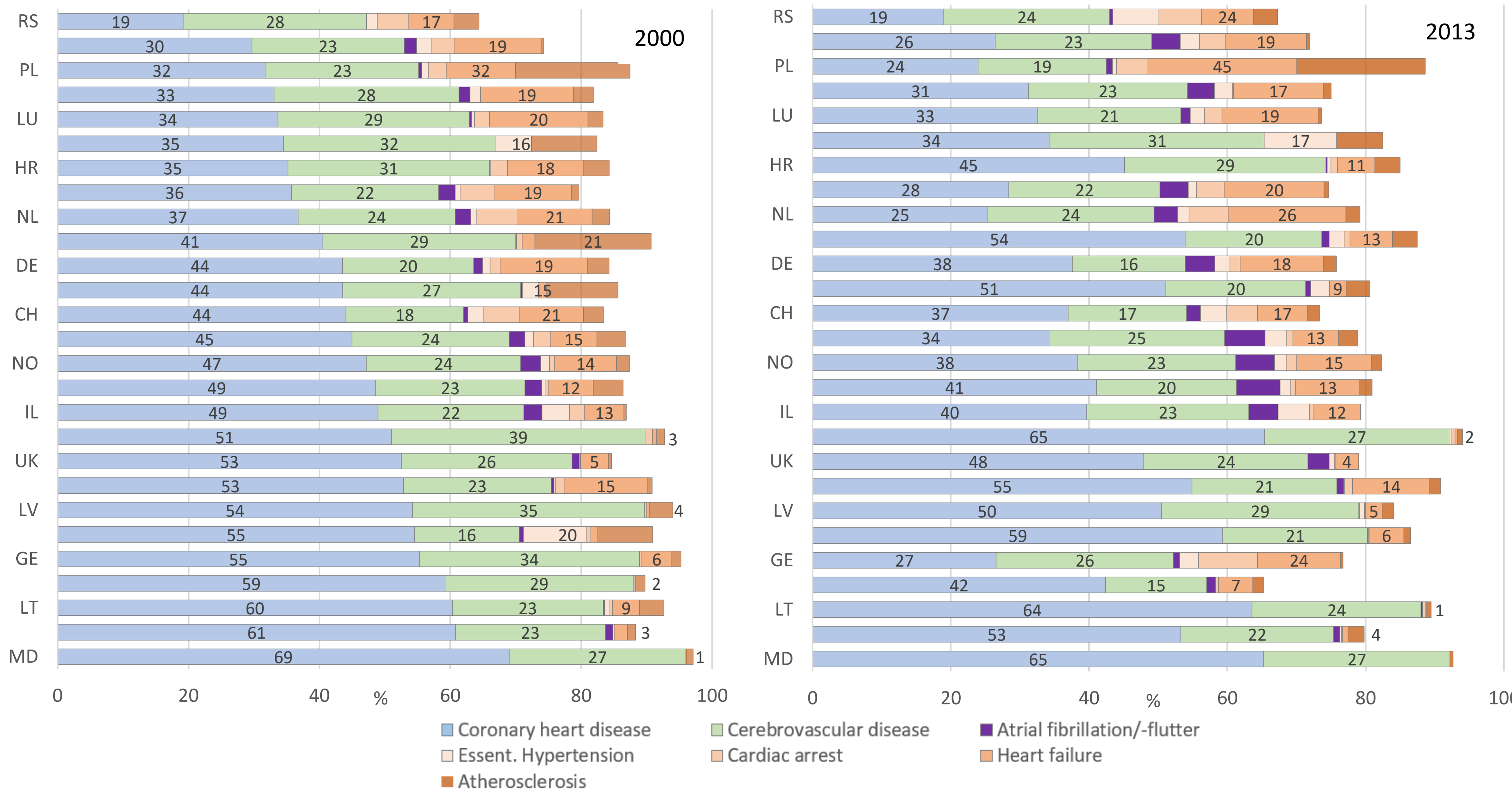

Fig S5: Among all cardiovascular deaths, proportion of deaths from selected cardiovascular diseases in populations ≥35 years in the WHO European Region in 2000 (left panel) and 2013 (right panel). Selected ill-defined cardiovascular causes (essential hypertension, cardiac arrest, heart failure and atherosclerosis) are indicated by reddish filling. Numbers represent the respective proportions. Numbers attached to the reddish bars indicate the proportion of all selected ill-defined causes combined; proportions less 0.5 are not given. Countries are ordered according to their proportion of deaths from coronary heart disease in 2000.

Abbr. BE: Belgium, CH: Switzerland, CZ: Czech Republic, DE: Germany, DK: Denmark, EE: Estonia, ES: Spain, FI: Finland, FR: France, GE: Georgia, HR: Croatia, HU: Hungary, IL: Israel, KG: Kyrgyzstan, LT: Lithuania, LU: Luxembourg, LV: Latvia, MD: Moldova, MT: Malta, NL: The Netherlands, NO: Norway, PL: Poland, RO: Romania, RS: Serbia, SE= Sweden, SK: Slovakia, UK: United Kingdom.

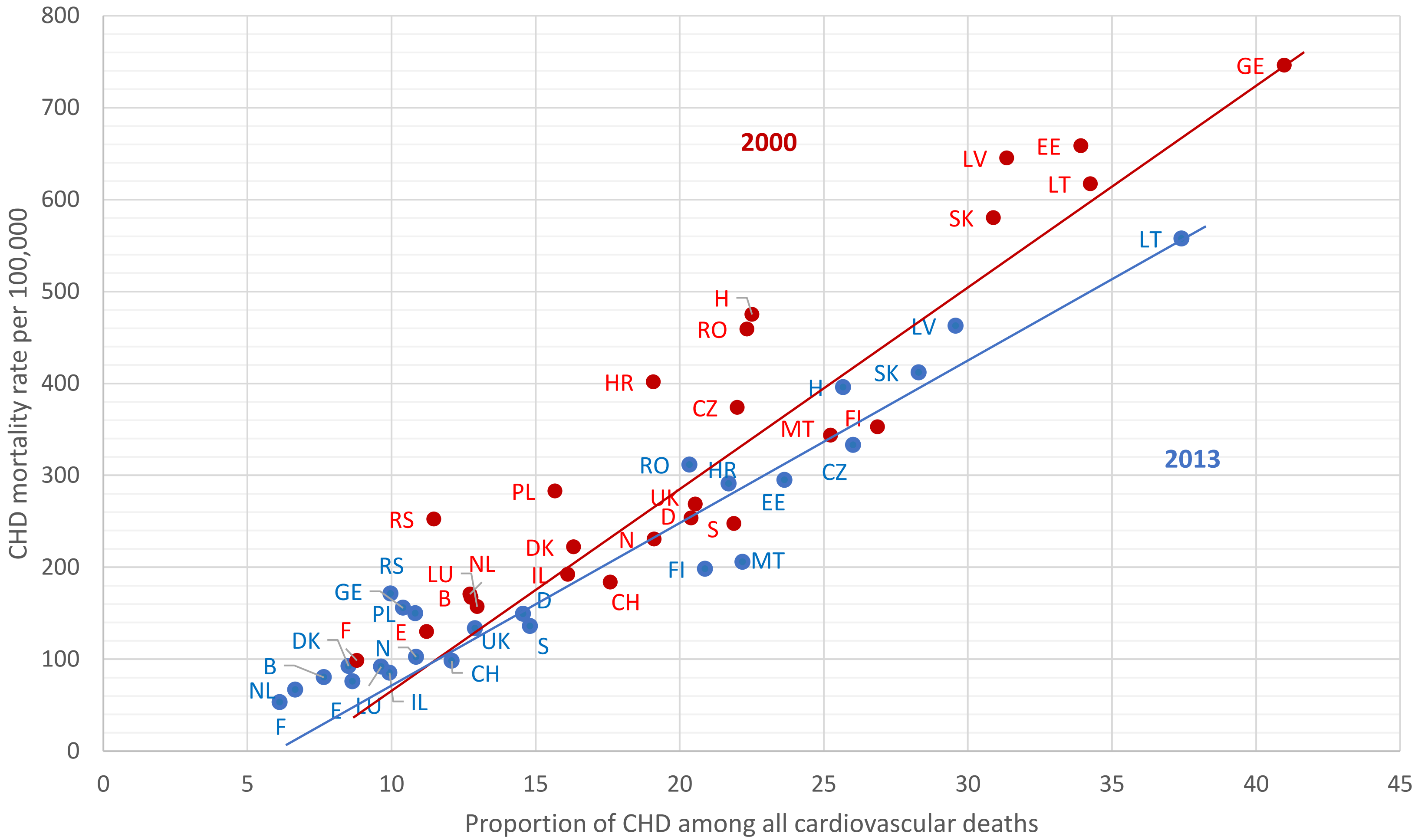

Fig S6. Association between proportion of deaths attributed to CHD among all cardiovascular deaths (x-axis) and age-standardized mortality rate for coronary heart disease (y-axis) for populations ≥35 years in countries of the WHO European region in 2000 (red dots) and 2013 (blue dots). Lines indicate population-weighted linear regression (R^2=0.74 in 2000 and R^2=0.82 in 2013).

Abbr. BE: Belgium, CH: Switzerland, CZ: Czech Republic, DE: Germany, DK: Denmark, EE: Estonia, ES: Spain, FI: Finland, FR: France, GE: Georgia, HR: Croatia, HU: Hungary, IL: Israel, KG: Kyrgyzstan, LT: Lithuania, LU: Luxembourg, LV: Latvia, MD: Moldova, MT: Malta, NL: The Netherlands, NO: Norway, PL: Poland, RO: Romania, RS: Serbia, SE= Sweden, SK: Slovakia, UK: United Kingdom.

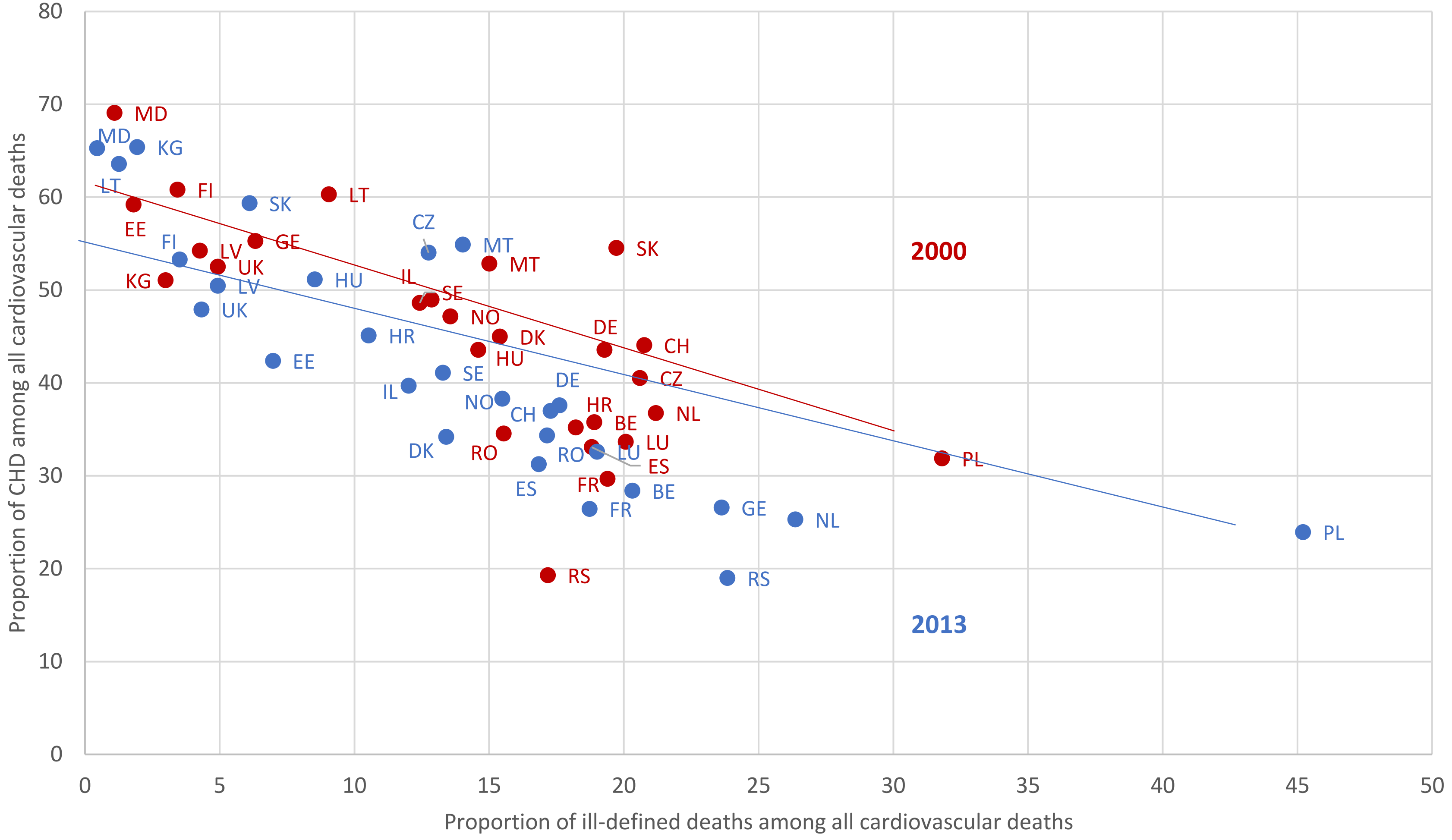

Fig S7. Association between proportion of ill-defined cardiovascular deaths (x-axis) and proportion of cardiovascular deaths attributed to CHD (y-axis) for populations ≥35 years in countries of the WHO European region in 2000 (red dots) and 2013 (blue dots). Lines indicate population-weighted linear regression.

Abbr. BE: Belgium, CH: Switzerland, CZ: Czech Republic, DE: Germany, DK: Denmark, EE: Estonia, ES: Spain, FI: Finland, FR: France, GE: Georgia, HR: Croatia, HU: Hungary, IL: Israel, KG: Kyrgyzstan, LT: Lithuania, LU: Luxembourg, LV: Latvia, MD: Moldova, MT: Malta, NL: The Netherlands, NO: Norway, PL: Poland, RO: Romania, RS: Serbia, SE= Sweden, SK: Slovakia, UK: United Kingdom
